# Protective effects of the *Francisella tularensis* Δ*pdpC* mutant against its virulent parental strain SCHU P9 in Cynomolgus macaques

**DOI:** 10.1038/s41598-019-45412-8

**Published:** 2019-06-24

**Authors:** Deyu Tian, Akihiko Uda, Yasushi Ami, Akitoyo Hotta, Eun-sil Park, Noriyo Nagata, Naoko Iwata-Yoshikawa, Akio Yamada, Kazuhiro Hirayama, Kozue Miura, Yuki Koyama, Mika Azaki, Shigeru Morikawa

**Affiliations:** 1 Laboratory of Veterinary Public Health, Department of Veterinary Medical Science, Graduate School of Agricultural and Life Sciences, The University of Tokyo, Yayoi 1-1-1, Bunkyo-ku, Tokyo 113-8657 Japan; 2 Department of Veterinary Science, National Institute of Infectious Diseases, Toyama 1-23-1, Shinjuku-ku, Tokyo 162-8640 Japan; 3 Division of Experimental Animal Research, National Institute of Infectious Disease, Gakuen 4-7-1, Musashimurayama-shi, Tokyo 208-0011 Japan; 4 Department of Pathology, National Institute of Infectious Diseases, Gakuen 4-7-1, Musashimurayama-shi, Tokyo 208-0011 Japan; 5 Major Track of Applied Veterinary Science, Doctoral Course of the United Graduate School of Veterinary Sciences, Gifu University, Yanagido 1-1, Gifu-shi, Gifu 501-1193 Japan; 6 Department of Integrated Science in Physics and Biology, College of Humanities and Sciences, Nihon University, Sakurajosui 3-25-40, Setagaya-ku, Tokyo 156-8550 Japan

**Keywords:** Bacterial infection, Live attenuated vaccines, Bacterial infection

## Abstract

Tularemia is a severe infectious zoonotic disease caused by *Francisella tularensis*. Although *F. tularensis* is considered to be a potential biological weapon due to its high infectivity and mortality rate, no vaccine has been currently licensed. Recently, we reported that *F. tularensis* SCHU P9 derived Δ*pdpC* strain lacking the pathogenicity determinant protein C gene conferred stable and good protection in a mouse lethal model. In this study, the protective effect of Δ*pdpC* was evaluated using a monkey lethal model. Two cynomolgus macaques (*Macaca fascicularis*) intratracheally challenged with the virulent strain SCHU P9 were euthanized on 7 and 11 days post-challenge after the development of severe clinical signs. The bacterial replication in alveolar macrophages and type II epithelial cells in the lungs would cause severe pneumonia accompanied by necrosis. Conversely, two animals subcutaneously immunized with Δ*pdpC* survived 3 weeks after SCHU P9 challenge. Though one of the two animals developed mild symptoms of tularemia, bacterial replication was limited in the respiratory organs, which may be due to a high level of humoral and cellular immune responses against *F. tularensis*. These results suggest that the Δ*pdpC* mutant would be a safe and promising candidate as a live attenuated tularemia vaccine.

## Introduction

Tularemia is a severe and sometimes fatal zoonotic disease caused by the Gram-negative intracellular bacterium *Francisella tularensis*. Based on pathogenic, biochemical, and geographical profiles, *F. tularensis* is classified into four subspecies: *tularensis*, *holarctica*, *mediasiatica*, and *novicida*^1^. *F. tularensis* subsp. *tularensis*, isolated predominantly in North America, is one of the most infectious bacteria with a minimum infective dose to human as low as 10 colony forming unit (CFU)^2–4^. If an appropriate antibiotic is not timely administered, the mortality rate is 5% to 30%^5^. *F. tularensis* subsp. *tularensis* is highly infectious and pathogenic, thus it is considered a potential threat as a biological weapon. However, at present, there is no suitable vaccine against tularemia.

To prevent human tularemia, attempts to develop a vaccine have employed a variety of strategies, and some vaccine candidates have been shown to be effective against tularemia in animal models^6^. The first vaccine for tularemia was a heat-killed *F. tularensis* produced by Lee Foshay in 1930^7^. During the Second World War, live attenuated vaccines were developed and found to be protective^8^. In 1956, ampoules of a viable mixture of attenuated tularemia vaccine were transferred to the United States from the Institute of Epidemiology and Microbiology Imeni N. F. Gamaleia (Gamaleia Institute) in the former Soviet Union by Shope^8^. From this mixture, a strain of the attenuated phenotype was selected, evaluated for safety, and designated as *F. tularensis* live vaccine strain (LVS)^9^. In human volunteers, the LVS was found to protect against the intracutaneous challenge of SCHU S4 but not against respiratory challenge^3,10^. More recently, various mutant strains based on LVS against *F. tularensis* (such as Δ*SodB*^11^ and Δ*dsbA*^12^), *F. novicida* (such as Δ*IglD*^13^ and Δ*iglB*^14^), and *F. tularensis* strain SCHU S4 (such as Δ*clpB*^15^ and Δ*ggt*^16^) were found to be attenuated with some protective capacity against the virulent strain SCHU S4 in mice or rats. However, there is currently no licensed vaccine against tularemia^17^. Thus, development of a safe and efficacious tularemia vaccine is still crucial.

Mouse, rat, rabbit, guinea pig, and macaque models have been applied in various tularemia vaccine studies^18^. Among these animal models to evaluate the efficacy of a vaccine, macaques develop clinical signs of pulmonary infection, quite similar to those in humans due to their close genetic backgrounds^19–21^. Tularemia outbreaks in cynomolgus (*Macaca fascicularis*)^22^ and rhesus (*Macaca mulatta*)^23^ macaques have also been reported. Previous studies have reported that macaques died 5 to 11 days post-challenge (dpc) with a virulent strain. The target organs in macaques and humans include the lung, liver, spleen, and lymph nodes^21,22,24^. Thus, a macaque model is well-suited to study the pathogenesis of tularemia and develop countermeasures in humans^3,10^. In fact, macaque models were used to evaluate the pathogenicity of tularemia pathogenicity in the early 1970s^24–28^ and to evaluate candidate tularemia vaccines in the 1960s^29–32^. The cynomolgus macaque is a better model of inhalation tularemia than the rhesus macaque^33^, as the pathology of cynomolgus macaques infected with aerosolized *F. tularensis* is similar to those observed in human inhalation tularemia^20^. The 50% lethal dose (LD_50_) of the virulent strain SCHU S4 in cynomolgus macaque is approximately 20 CFU via aerosol infection^10,33^.

In our previous study, we established a novel knockout mutant strain with an attenuated phenotype by intron insertion into the pathogenicity determinant protein C gene (*ΔpdpC*) of *F. tularensis* strain SCHU P9, which showed good protection against 100 LD_50_ virulent strain challenge in a mouse model^34^. In the present study, the Δ*pdpC* mutant was further evaluated as a candidate live attenuated vaccine against tularemia in cynomolgus macaques challenged with a massive lethal dose (10^6^ CFU) of virulent strain SCHU P9 because the respiratory infection is thought to cause the most serious consequences of *F. tularensis* if used as a biological weapon.

## Results

### Vaccination with attenuated *F. tularensis* strain ∆*pdpC* protected cynomolgus macaques from a lethal challenge of *F. tularensis*

**Figure 1:**
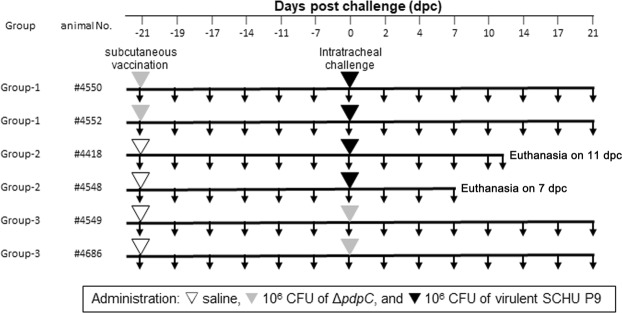
Schematic outline for of Vaccination and challenge. Two animals in Group 1 (G1: #4550 and #4552) were subcutaneously vaccinated with 10^6^ CFU of ∆*pdpC* (gray inverted triangle) at −21 dpc, whereas four animals in Group 2 (G2: #4118 and #4548) and Group 3 (G3: #4549 and #4686) were similarly administrated the same volume of saline (white inverted triangle). At 0 dpc, the four animals in Groups 1 and 2 were challenged intratracheally with 10^6^ CFU of virulent SCHU P9 (black inverted triangle), whereas the two animals in Group 3 were similarly administrated ∆*pdpC* (gray inverted triangle). The black arrows indicate the schedule of blood collection and measurement of body weight and temperature on −21, −19, −17, −14, −11, −7, 0, 2, 4, 7, 10, 14, 17, and 21 dpc. Two animals in G2 (#4118 and #4548) were euthanized at 11 and 7 dpc because of severe clinical signs. All animals in Groups 1 and 3 the survived the observation period were euthanized at 21 dpc.

**Figure 2:**
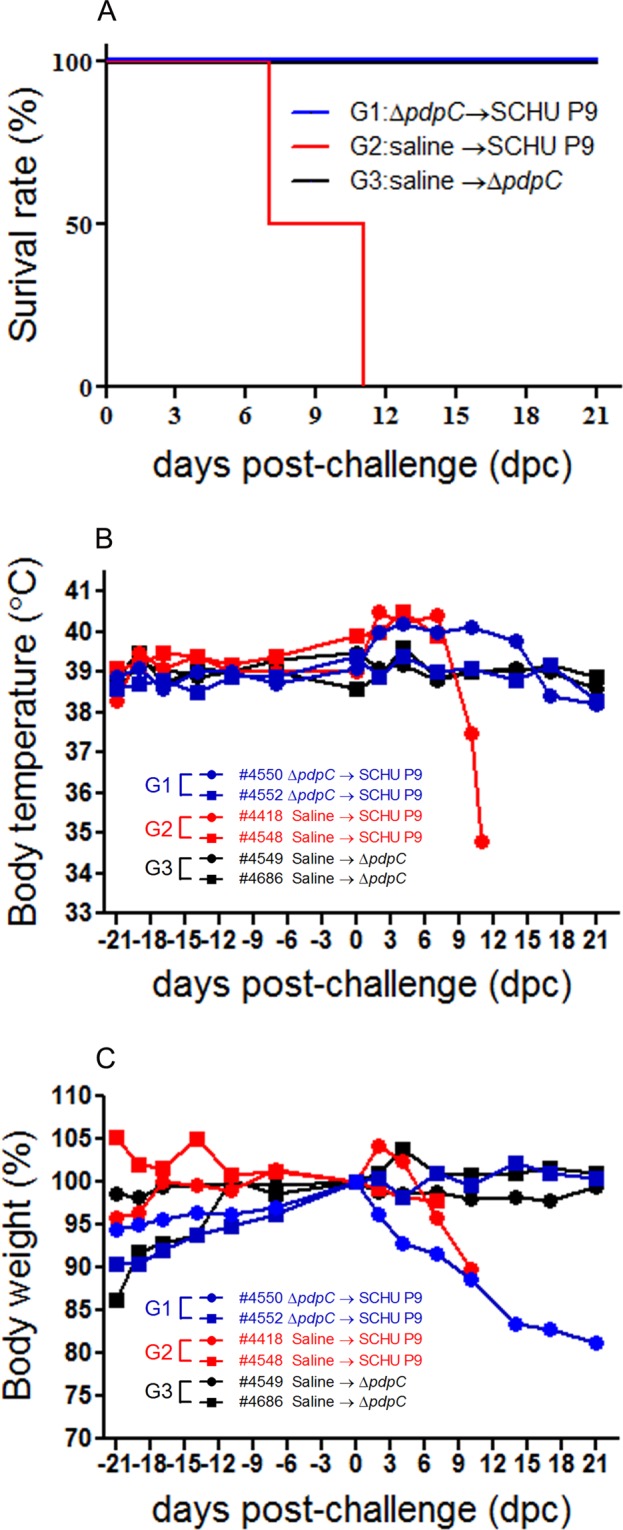
Protection from lethal challenge in cynomolgus macaques immunized with ∆*pdpC*. Six healthy adult male cynomolgus macaques were randomly classified into three groups. Two animals in Group 1 were subcutaneously immunized with 10^6^ CFU of mutant ∆*pdpC* at −21 dpc, whereas the other four animals in Groups 2 and 3 were subcutaneously injected with saline. After 3 weeks, the animals in Groups 1 and 2 were intratracheally challenged with 10^6^ CFU of the virulent strain SCHU P9 at 0 dpc, and two animals in G3 were intratracheally challenged with 10^6^ CFU of ∆*pdpC*. All animals were monitored every day during the experimental period. The survival rates (**A**), body temperatures (**B**), and body weights (**C**) of the animals in Groups 1 (blue), 2 (red), and 3 (black) are shown.

Two animals in Group 2 (#4418 and #4548) were mock-vaccinated and then challenged with the virulent strain SCHU P9 following the schedule shown in Fig. 1. Both developed severe clinical signs of tularemia, which included a cough, diminished appetite, decreased activity, depression, and a recumbent position in the cage with feeble stimulation responses. Therefore, these animals were considered to be reached the humane endpoint. The animal #4548 was euthanatized at 7 dpc. The body temperature (Fig. 2B) and the body weight (Fig. 2C) of the other animal (#4418) had decreased sharply after the challenge and was euthanatized at 11 dpc.

Two animals in Group 1 (#4550 and #4552) were vaccinated with strain ∆*pdpC* and then challenged with strain SCHU P9. One (#4550) of the animals developed a fever after the challenge but recovered after 2 weeks (Fig. 2B). Animal #4550 lost a significant amount of body weight (Fig. 2C), but its general condition, including appetite, was normal. Animal #4552 showed no clinical signs during the experimental period and survived until the end of the experiment (Fig. 2A). The animals in Group 3 (#4549 and #4686) were mock-vaccinated and then challenged with ∆*pdpC* and showed no clinical symptoms during the experimental period (Fig. 2).

### Necropsy findings

**Figure 3:**
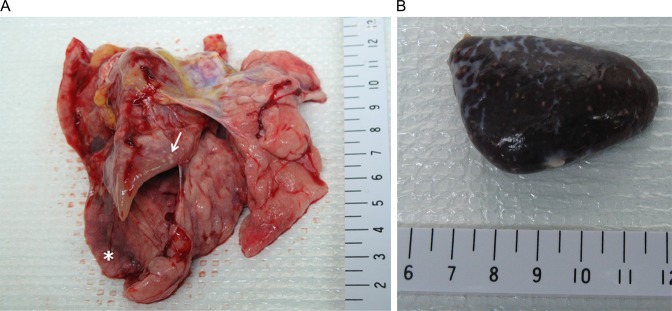
Macroscopic pathologies. (**A**) The lungs of two animals after virulent SCHU P9 challenge (Group 2) had hemorrhages (asterisk) and white lesions (arrow). (**B**) The spleen had blunt edges, and the spleen of animal #4418 had white lesions.

Two animals (#4418 and #4548) in Group 2, challenged with the virulent strain SCHU P9 without vaccination, showed obvious clinical signs of tularemia, including ascites and hydrothorax (Supplementary Table 1). The lungs of both animals showed severe pathology of tularemia, hemorrhage (asterisk), and white lesions (arrow) (Fig. 3A). The spleens of both animals had blunt edges and that of animal #4418 had white lesions (Fig. 3B). On the other hand, two animals (#4549 and #4686) in Group 3, which were inoculated with only strain ∆*pdpC*, showed no gross abnormalities in the organs although enlargement of the cervical, axillary, and inguinal lymph nodes were observed (Supplementary Table 1). One vaccinated animal (#4552) challenged with the virulent strain SCHU P9 in Group 1 had no clinical signs or gross pathological findings, whereas the other vaccinated animal (#4550) had focal lesions in the right lung.

### Histopathological findings

**Figure 4:**
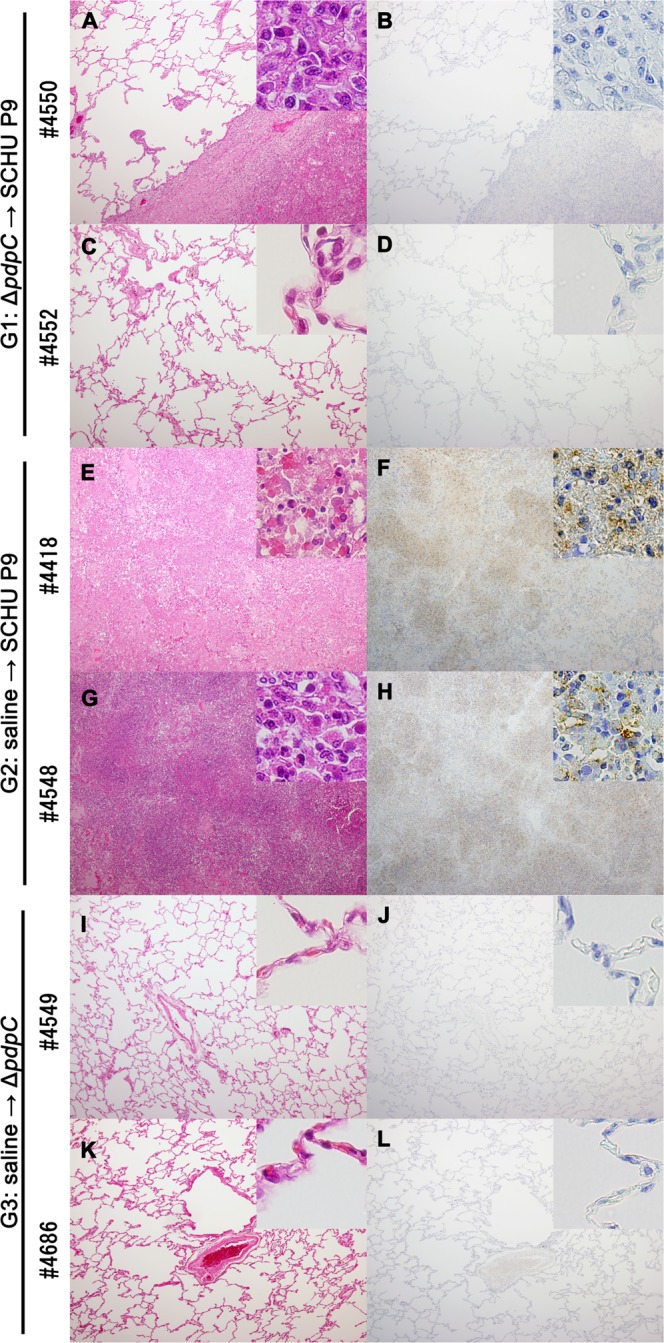
Histopathological analysis of lung in immunized cynomolgus macaques after the challenge. The lungs of the euthanized animals in Groups 1, 2, and 3 were fixed in 10% formalin neutral buffer solution, embedded in paraffin, sectioned, and stained with hematoxylin and eosin ((**A,C,E,G,I,K**); original magnification ×4 and insert original magnification ×40). Similarly, the slides were immunohistochemically stained to detect the *F*. *tularensis* LPS antigen ((**B,D,F,H,J,L**); original magnification ×4 and insert original magnification ×40).

Two animals (#4418 and #4548) in Group 2 showed obvious histopathological features of tularemia. The lung structures were almost destroyed, and neutrophils, macrophages, and purulent necrotic substances filled the lungs. Severe lung abscesses and purulent lesions accompanied with necrosis extended from the end of the bronchioles to the alveoli (Fig. 4E,G). Necrosis was also found in the livers (Supplementary Table 1), and lymphocytopenia was observed in the spleens (data not shown).

Of the two animals in Group 1 (#4550 and #4552), no histopathological lesions were observed in the organs of animal #4552 at 21 dpc (Fig. 4C), whereas the right lung of animal #4550 contained histopathological lesions, which were less severe than those of the animals in Group 2 (Fig. 4A). The abscesses were composed of epithelial cells, fibroblasts, and infiltrated macrophages and neutrophils, that were completely isolated by a thick fibrous hyperplasia in the lung of animal #4550 (Fig. 4A). Animals in Group 3 (#4549 and #4686) had no histopathological lesions in the lungs (Fig. 4I,K) or other organs (data not shown).

**Figure 5:**
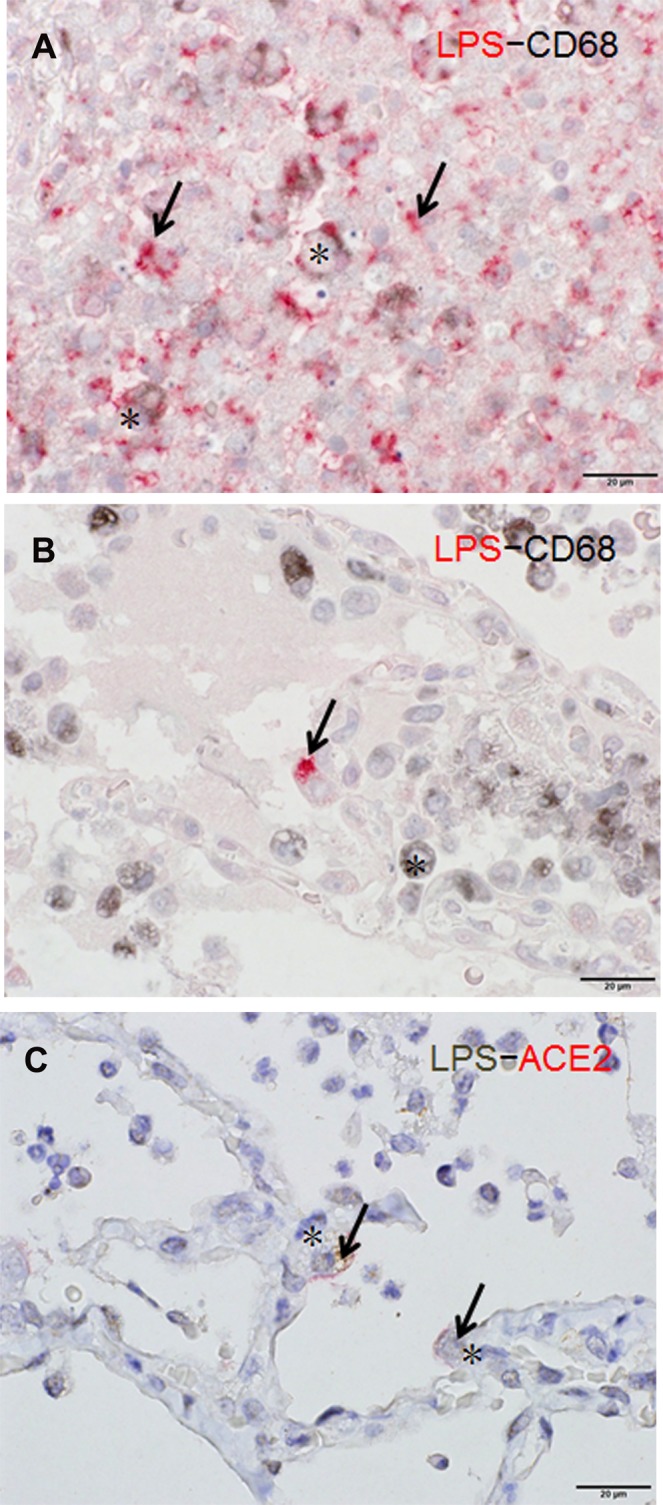
The detection of the infected macrophages and AT-II cells in cynomolgus macaque. The lung of animal #4458 in Group 2 was immunohistochemically stained to identify the infected macrophages and AT-II cells (original magnification ×100). (**A,B**) The lung was stained with anti–*F. tularensis* LPS (red) and anti-CD68 (brown) Abs to detect infected macrophages. An abundance of LPS antigens was detected in macrophages stained with the anti-CD68 Ab (asterisks) and other cells (arrows). (**C**) When the lungs were double stained with anti–*F. tularensis* LPS antigen (brown) and anti-ACE2 (red), the AT-II cells were detected altogether with *F. tularensis* LPS antigen (arrows).

Immunohistochemical analysis using anti–*F. tularensis* LPS monoclonal Ab revealed an abundance of LPS antigen-positive foci, which were considered to be *F. tularensis*, in the lung lesions of the animals in Group 2 (Fig. 4F,H). *F. tularensis* LPS antigen-positive foci were demonstrated in the restricted necrotic lesions of the right lung of animal #4550, whereas no antigens were found in the lesion-free areas (Fig. 4B). Since *F. tularensis* is intracellular bacteria and the LPS antigens were found in the cells in the lung lesion, double immunohistochemical staining of the lung lesions was conducted with anti–*F. tularensis* LPS monoclonal Ab and an Ab against the cell marker. The results showed that bacteria invaded not only macrophages (Fig. 5A) but also other cells (Fig. 5B), which were shown to be ACE2 positive alveolar epithelial type II (AT-II) cells (Fig. 5C).

### Bacterial burden

**Figure 6:**
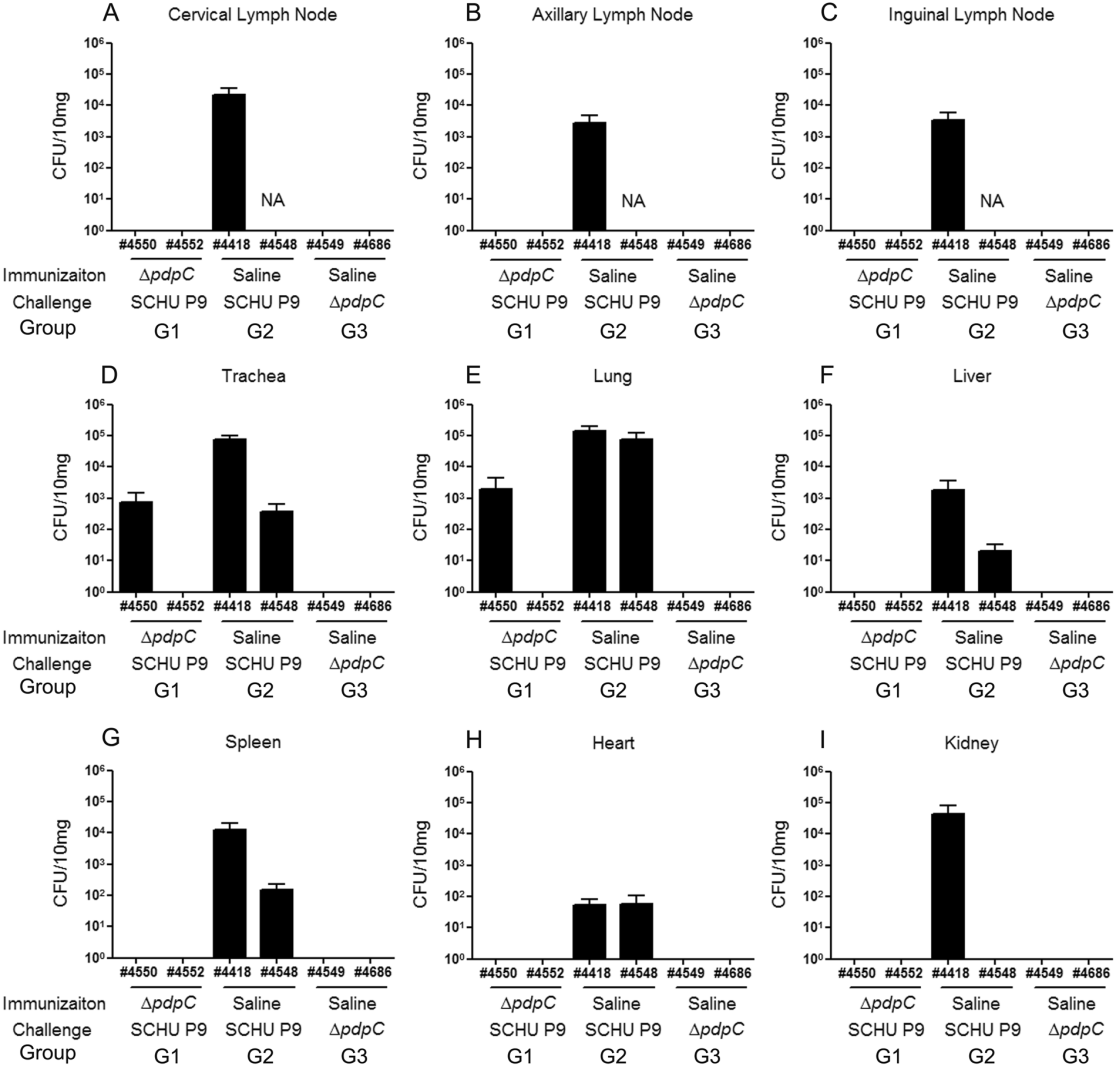
The bacterial replication limited in the respiratory organ of the immunized cynomolgus macaques after the challenge. Three samples of each organ (approximate 100 mg/piece) were collected from the sacrificed animals in Group 1 (G1, animal #4550 and #4552), Group 2 (G2, animal #4418 and 4548), and Group 3 (G3, animal #4549 and #4686), minced, and homogenized in RPMI 1640. The serially diluted homogenates were cultured on Eugon chocolate agar plates in duplicate. CFU in each minced sample was calculated from an average number of colonies in duplicate. The mean ± SD of CFU in three minced samples derived from each cervical lymph node (**A**), axillary lymph node (**B**), inguinal lymph node (**C**), trachea (**D**), lung (**E**), liver (**F**), spleen (**G**), heart (**H**), and kidney (**I**) are shown. NA, not applicable.

The cervical, axillary, and inguinal lymph nodes, and the tracheas, lungs, livers, spleens, hearts, and kidneys were submitted for bacterial culture at the time of postmortem examination except for the lymph nodes of animal #4548 in Group 2, which were atrophied and a sufficient amount for bacterial culture could not be obtained. Bacteria were detected in the lymph nodes, tracheas, lungs, livers, spleens, hearts, and kidneys of the animals in Group 2 (Fig. 6). *F. tularensis* was also detected in pleural effusion of the animals in Group 2 that had symptoms of tularemia (data not shown). On the contrary, of the two animals in Group 1, which were immunized with the mutant strain ∆*pdpC* and challenged with the virulent strain SCHU P9, *F. tularensis* was detected only in the trachea and lung of animal #4550 (Fig. 6). *F. tularensis* was not detected in any of the organs and tissues of the animals in Group 3 (Fig. 6).

### Ab responses in animals infected with *F. tularensis*

**Figure 7:**
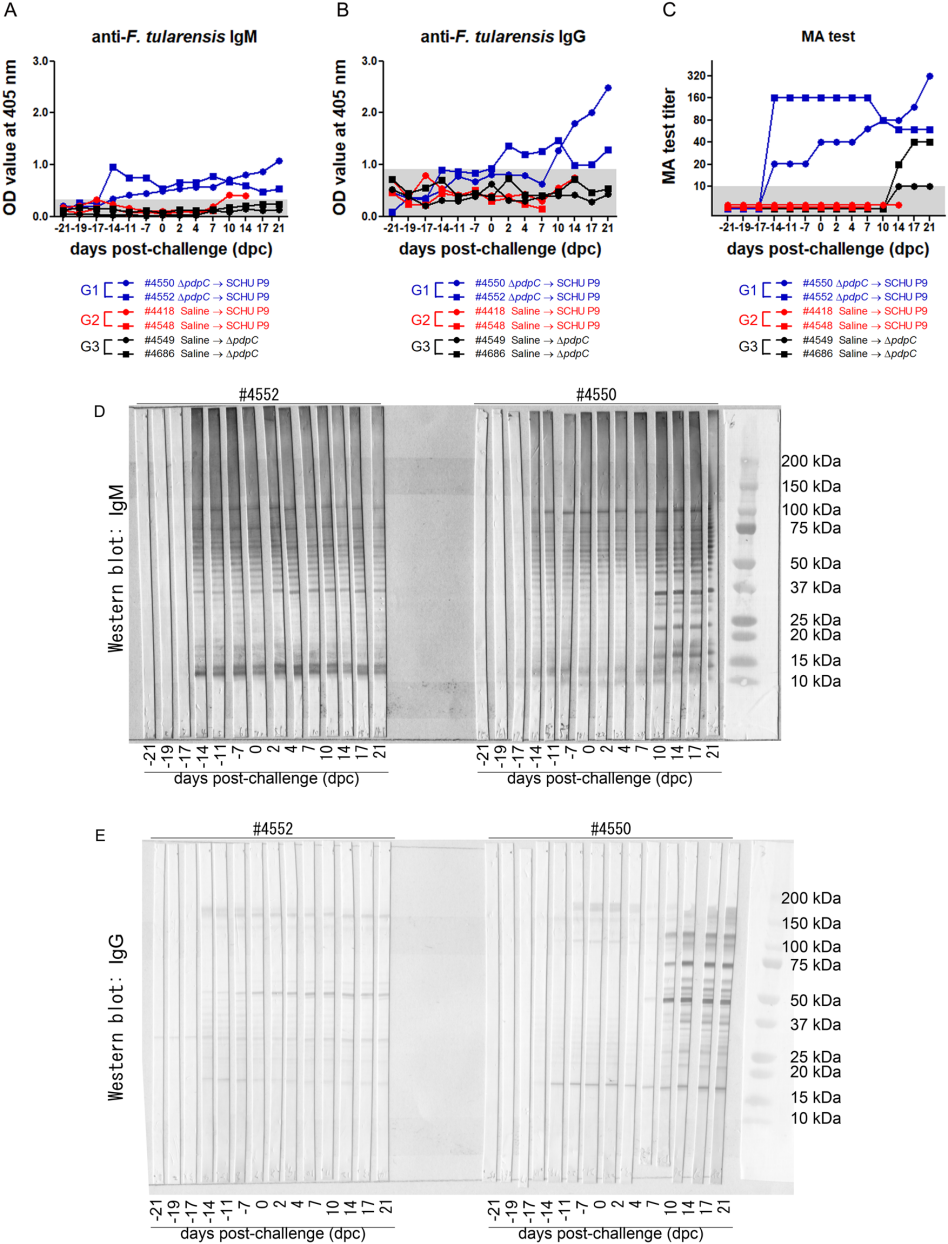
Confirmation of the high Ab titers against *F. tularensis* in cynomolgus macaques after immunization. Blood samples were obtained according to the schedule shown in Fig. 1. (**A,B**) The titers of specific IgM (**A**) and IgG (**B**) Abs against *F. tularensis* were measured with an ELISA. The average OD_405_ value are shown. The gray background indicates the average OD_405_ + 2 SD in Groups 2 and 3 at −21, −19, −17, −14, −11, −7, and 0 dpc without bacteria. (**C**) The MA test was conducted to confirm the ELISA results. The average dilution factors are shown. The gray background indicates an undetectable level of specific Abs. (**D,E**) The specific Abs in the immunized cynomolgus macaques were confirmed by western blotting. Blood samples were obtained according to the schedule shown in Fig. 1. Whole bacterial lysate of strain SCHU P9 was electrophoresed on an SDS-polyacrylamide gel and electrophoretically transferred to a PVDF membrane. The partially dried PVDF membrane was cut into small strips that were incubated with sera diluted 1000-fold. Subsequentially, the strips were incubated with HRP-conjugated rabbit anti-monkey IgG or HRP-conjugated goat anti-monkey IgM and then visualized by incubation with DAB. Molecular weight markers are shown in the right lane. The individual dried strips originated from the antigen transferred PVDF membrane were reacted with periodically collected serum samples of the animals and they were aligned on white papers. The grayscale images of the papers were scanned using DocuCentre-IV C2263 (Fuji Xerox, Tokyo, Japan) at superfine resolution (600 dpi) and saved as JPEG format files. The image processing was done using Photoshop CS2 (Adobe Inc, San Jose, CA, USA) to crop the images but brightness and contrast adjustment were not done. The full-length blots are shown in the figure.

Specific IgM and IgG Abs against *F. tularensis* antigen were measured with an ELISA as previously reported^35^. The mean + 2 standard deviations (SD) of the OD_405_ value in uninfected animals (Group 2 and 3 at −21, −19, −17, −14, −11, −7, and 0 dpc) were calculated as the upper limit of the non-response ranges against *F. tularensis* (Fig. 7A,B). Anti–*F. tularensis* IgM was elicited at −14 dpc in animal #4552 in Group 1, whereas IgM response was weaker in animal #4550 in Group 1 (Fig. 7A). IgG Abs of animals #4552 and #4550 in Group 1 were strongly induced at 2 and 10 dpc, respectively (Fig. 7B).

Two animals in Group 2 had no Ab response upon SCHU P9 infection, except animal #4418, which showed weak responses before becoming moribund. Two animals in Group 3 showed feeble Ab responses (Fig. 7A,B).

Ab responses were also measured by the MA Ab (MA) test. Ab responses were detected in the animals in Group 1 at −14 dpc, and Ab titers had increased upon SCHU P9 challenge in animal #4550 (Fig. 7C). The animals in Group 3 also showed elevated Ab titers at 14 dpc. On the other hand, Abs were not detected in the animals in Group 2 (Fig. 7C).

Ab responses were also examined by WB analysis using *F. tularensis* lysates as antigens. WB analysis revealed that the sera of the animals in Group 1 showed specific ladder bands, with molecular weights of 15–100 kDa for IgM and 17–200 kDa for IgG at 7 dpc (Fig. 7D). The serum of animal #4552 showed steady levels of bands even before SCHU P9 challenge, whereas the serum of animal #4550 showed significantly strong bands at 10 dpc. These different Ab responses were in accordance with those demonstrated by the ELISA and MA test. The sera of the animals in Group 2 had no apparent ladder bands until 7 dpc with SCHU P9. Weak ladder bands were detected at 10 dpc in the serum of animal #4418, which died at 11 dpc (data not shown). The sera of the animals in Group 3 showed weak ladder bands at 14 dpc with strain ∆*pdpC* (data not shown).

### IFN-γ secreting T cells evaluated by ELISpot assay

**Figure 8:**
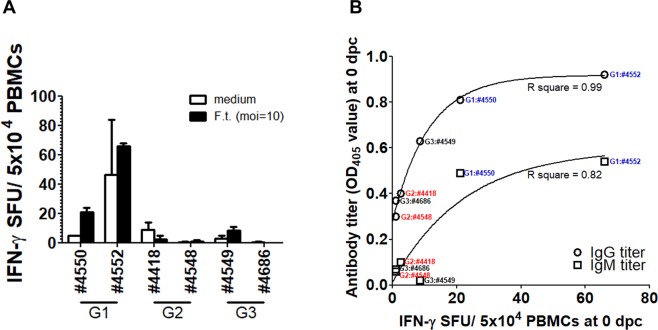
ELISpot assay on PBMCs. (**A**) PBMCs were prepared from the monkey blood specimens obtained at 0 dpc. IFN-γ secreting T cells in PBMCs of the individual animas were measured by ELISpot Primate IFN-γ Kit (R&D). Fifty thousand PBMCs were incubated in RPMI 1640 medium containing 10% FBS and 50 µg/ml gentamycin for 24 h at 37 °C with and without SCHU P9 as stimulus. The experiments were performed in duplicate. The mean ± SD of spot-forming units (SFU)/5 × 10^4^ cells are shown. (**B**) Scatter plots of the significant correlation at 0 dpc between Ab titers measured by ELISA and specific IFN-γ -secreting T cells measured by ELISPOT are shown. IgM and IgG titers are indicated by square and circle plots, respectively. Curves are fit to a one-phase association curve using Graphpad Prism.

Specific IFN-γ secreting T cells against *F. tularensis* antigen were evaluated with the ELISpot assay using the peripheral blood mononuclear cells (PBMCs) at 0 dpc of the animals (Fig. 8A). Upon stimulation with SCHU P9 at a moi = 10, the means of spot forming units (SFU) in animal #4450 and #4552 in Group 1 were 21 and 66, respectively, which were significantly higher than those in Group2 and 3. Thus, IFN-γ secreting T cells were considered to be induced in PBMCs of the vaccinated animals in G1.

The correlation between Ab titers measured by ELISA and specific IFN-γ secreting T cells measured by ELISPOT were analyzed using data that were observed at 0 dpc. As shown in Fig. 8B, both Ab titers and specific IFN-γ-secreting T cells in vaccinated animals #4450 and #4552 in Group 1 were higher than those in other animals were not vaccinated. In addition, a one-phase association curve fitted the data well for all IgG and IgM plots, with R square values of 0.99 and 0.82, respectively.

## Discussion

*F. tularensis* subspecies *tularensis* is a highly infectious pathogen and causes the lethal disease tularemia. In addition to natural infection of humans and animals, its potential to be used as a biological weapon highlights the necessity to develop effective vaccines to protect against this pathogen. Among the many methods applied to this disease, attenuated live vaccines are often applied^18,36^. A previous study reported that the ∆*pdpC* mutant strain had the potential to protect mice from lethal intranasal challenge with a virulent strain via subcutaneous immunization^34^. The *pdpC* gene of the virulent strain SCHU P9 was disrupted by group II intron insertion using the TargeTron gene knockout system^37,38^ to generate the Δ*pdpC* mutant strain^39^. Thus, the genetic stability of strain ∆*pdpC* was confirmed by *in vivo* passages in mice for 10 generations^34^. These data suggest that the mutant strain Δ*pdpC* is a promising vaccine candidate for tularemia. To further evaluate the possibility of Δ*pdpC* as a candidate tularemia vaccine, a lethal cynomolgus macaque model was employed in this study to analyze a protective potential of Δ*pdpC*.

Six cynomolgus macaques were used to assess the protective effects of the mutant strain ∆*pdpC* against intratracheal challenge with the virulent strain SCHU P9. The majority of animal experiments so far reported were initiated with the highly virulent strain SCHU S4, a variant of the virulent strain SCHU, which was initially isolated by Foshay from an ulcer of American patient in 1941^40,41^. The virulent strain SCHU was obtained by the Ohara Research Laboratory, Ohara General Hospital (Fukushima, Japan) from the Rocky Mountain Laboratory of the National Institute of Allergy and Infectious Diseases (Hamilton, MT, USA) in 1958^42^. Then, the virulence of strain SCHU was attenuated by 373 passages on pig liver glucose hemoglobin agar medium over a period of 30 years^42^. The attenuated SCHU strain was kindly provided by Dr. H. Fujita (Ohara Research Laboratory) in 2002. In Japan, it is difficult to obtain strain SCHU S4 from foreign laboratories because the transport of the bacterium is highly regulated under the Japanese infectious disease control laws. We recently isolated a virulent SCHU strain (referred to as SCHU P9) from the attenuated SCHU strain after 9 passages in mice^39^. Strain SCHU P9 has the same parental original strain as strain SCHU S4, and its virulence in mice was shown to be comparable to that of SCHU S4^39^.

In the study, cynomolgus macaques were inoculated intratracheally with a massive dose (10^6^ CFU) of the virulent strain SCHU P9 to mimic a route of infection during a bioterror attack. The LD_50_ of the virulent strain SCHU P9 was not determined in cynomolgus macaques because of the limited number of animals in the experiment. Our previous study showed that the LD_50_ of the virulent strain SCHU P9 in mice was comparable to that of strain SCHU S4; thus, the LD_50_ of the virulent strain SCHU P9 in cynomolgus macaques was considered to be similar to that of strain SCHU S4. An earlier study reported that the LD_50_ of strain SCHU S4 in rhesus macaques was between 14 and 4,447 CFU based on aerosol particle size^28^, whereas that in cynomolgus macaque was approximately 20 CFU^10,33^ via the aerosol route. Based on these reports, an intratracheal infective dose of 10^6^ CFU of SCHU P9 of no less than 1,000 LD_50_ was used in this study. Two monkeys in Group 2 that were challenged intratracheally with 10^6^ CFU of SCHU P9 without prevaccination developed severe tularemia and were euthanized at 7 or 11 dpc. Two monkeys in Group 1 that were vaccinated subcutaneously with the mutant strain ∆*pdpC* had survived for 21 dpc with the virulent strain SCHU P9, strongly indicating that subcutaneous vaccination with strain Δ*pdpC* is effective against lethal infection with a virulent strain of *F. tularensis*.

Pneumonic tularemia is naturally rare, but yet the most severe form of tularemia. As a potential agent of bioterrorism, the animals in this study were experimentally inoculated with *F. tularensis* intranasally or intratracheally. After inoculation, the bacteria arrive in the alveolar airspace and are immediately phagocytosed by alveolar macrophages and inactivated in phagolysosomes. However, *F. tularensis* can replicate efficiently in macrophages. In addition, Faron *et al*. recently demonstrated that virulent *F. tularensis* strains can efficiently replicate in alveolar AT-II cells both *in vitro* and *in vivo*^43–45^. Therefore, we tried to confirm the infection of alveolar macrophages and AT-II cells in the lungs of cynomolgus macaques. For immunohistochemical analysis of cynomolgus macaque, CD68, which is expressed specifically by monocytes, is often used to identify macrophages. AT-II cells in the lungs, which can be identified using Abs against angiotensin-converting enzyme 2 (ACE2), play a key role in the homeostasis in the renin–angiotensin system. In this study, the infection of alveolar macrophages and AT-II cells in cynomolgus macaque were confirmed using Abs against CD68 and ACE2, respectively, accompanied with Abs against *F. tularensis* LPS. The infected macrophages, which were double stained with anti-CD68 and anti–*F. tularensis* LPS Abs, were found in the lungs of the monkeys in Group 2 (Fig. 5A). Simultaneously, the infected AT-II cells that were double stained with anti-ACE2 and anti–*F. tularensis* LPS Abs were also observed in the lungs (Fig. 5C). These findings were first confirmed in nonhuman primates and strongly suggest that *F. tularensis* efficiently infects and replicates in AT-II cells after respiratory inoculation *in vivo*, as reported by several researchers^43–45^. Thus, it is likely that infected AT-II cells, as well as the infected macrophages, play a crucial role in the development of severe tularemia pneumonia.

*F. tularensis*-specific Abs were shown to play an important role to protect the host from *F. tularensis* infection^46,47^. In the present study, IgM and IgG ELISAs showed that *F. tularensis*-specific Abs were elicited in the animals in Group 1 after subcutaneous vaccination with strain ∆*pdpC* and intratracheal challenge with the virulent strain SHCU P9 (Fig. 7A,B). The induction of *F. tularensis*-specific Abs was also detected with the MA test (Fig. 7C) and WB analysis (Fig. 7D,E). It has been reported that when infected by *F. tularensis* by inhalation, serum Abs contribute to protecting against tularemia by preventing the spread of bacteria from the lungs to the liver and spleen while facilitating rapid bacterial clearance in the lung, which offers a considerable survival advantage^46,48^. The Ab titer in animal #4552 had increased after vaccination with strain ∆*pdpC*, as compared to animal #4550. Animal #4552 had no symptoms of tularemia, whereas the body weight of animal #4550 had decreased (Fig. 2C), suggesting that the stronger Ab response induced in animal #4552 contributed to protection against tularemia. The difference in protective immunity between these two animals may be due to differences in genetic backgrounds. In fact, vaccine testing in humans showed different protection effects among individual volunteers from asymptomatic to mild to severe^3,10,49^. In animal #4550, however, although all bacteria were not cleared from the trachea and lungs (Fig. 6), bacterial growth was limited in these organs, indicating a protective effect inhibiting the spread of the bacteria to other organs and tissues.

The protective effect of Ab generally supports the neutralization of bacterial infectivity and the lysis of the bacterial membrane by the complement system. However, *F. tularensis* is a facultative intracellular bacterium that can replicate in multiple types of eukaryotic cells including phagocytes such as macrophages and dendritic cells. Since the Ab is not able to directly react with the intracellular bacteria, the cellular immune responses might be also important to restrict the intracellular replication in *F. tularensis*, as well as humoral immune responses. For example, cytotoxicity T cells (CTL) playing a central role in cellular immune responses were indispensable for the escape from lethal infection of intracellular bacteria such as *Listeria monocytogenes*^50^ and *Mycobacterium tuberculosis*^51^. Several reports have shown that IFN-γ and tumor necrosis factor alpha (TNF-α) that could evoke cellular immune responses were indispensable to prevent lethal infection in *F. tularensis*^52–54^. However, it was uncertain that CTL could contribute to restricting the bacterial replication in *F. tularensis*. In this study, PBMCs collected at 0 dpc (before challenge) were applied to IFN-γ ELISpot assay (Fig. 8A). The abundant IFN-γ secreting T cells were detected in the PBMCs of the animals #4550 and #4552 in Group 1. Especially, the highest level of IFN-γ secreting T cells in PBMCs and the Ab responses were induced in the animal #4552 among the six animals (Fig. 8B). Since animal #4552 did not show any clinical signs at all (Fig. 2), it might be suggested that the synergy between cellular and humoral immune responses contributed to the forceful prevention from the lethal challenge of SCHU P9.

The high levels of specific Ab responses were often observed at 3 weeks post-vaccination in a tularemia mouse model, as previously reported^11,34^. Similarly, because high levels of MA test titers in two animals in Group 1 (animal #4550 and #4552) were confirmed at 3 weeks post vaccination in this study, the two animals were intratracheally challenged with virulent strain SCHU P9 at 3 weeks post-vaccination. The specific T cells stimulated by the strain SCHU P9 antigen were confirmed in animals #4450 and #4552 in Group 1 by performing the ELISPOT assay. Moreover, abundant IFN-γ-secreting T cells without the stimulation were also shown in the PBMCs of animal #4552 in Group 1 (Fig. 8A) because of the elevated immune responses that continued for up to 3 weeks post vaccination. These findings suggest that the elevated immune response contributed to survival from the lethal challenge in animal #4552 in Group 1. Conversely, it would have been preferable to have increased the time until the challenge to evaluate the acquired immune responses in accordance with previous reports of macaques that were challenged with the virulent strain at 1 and 2 months post vaccination^13,30^.

The route of vaccination also influences the immune response against invading pathogens^55^. The results of this study demonstrated that *F. tularensis*-specific Ab responses after inoculation with strain ∆*pdpC* differed between animals inoculated subcutaneously (Group 1) and those inoculated intratracheally (Group 3). The animals in the two groups were inoculated with the same dose of strain ∆*pdpC*, whereas subcutaneous inoculation with strain ∆*pdpC* induced higher Ab responses than intratracheal inoculation. Thus, an optimal route of administration, as well as suitable strain for the vaccine, should be considered to induce better protection against tularemia.

It has been reported that fever is a common clinical sign of tularemia in humans^3,10^ and squirrel monkeys^56^. In this study, fever was observed in the animals in Group 2 upon challenge with the virulent strain SCHU P9 (Fig. 2B). On the other hand, one (#4550) of the animals in Group 1 developed a fever after challenge but had recovered after 2 weeks, whereas the other (#4552) did not develop a fever (Fig. 2B). Also, the two animals in Group 3 had no fever. These results demonstrated that strain ∆*pdpC* is safe and effective against lethal tularemia in cynomolgus macaques.

*F. tularensis* LVS is used as the gold standard to evaluate the efficacy of vaccine candidates to protection against lethal infection of *F. tularensis* but it was not used in the present study because the number of available animals was limited. Thus the vaccine efficacy between LVS and Δ*pdpC* was not compared directly. In addition, the data could not be evaluated statistically because of the limited number of animals included in the study cohort. Although this was a preliminary study, our data would be useful for future experiments with non-human primate models of tularemia.

In conclusion, strain ∆*pdpC* was safe and efficacious against a lethal challenge of *F. tularensis* in cynomolgus macaques, indicating that strain ∆*pdpC* is a good candidate for a live attenuated vaccine against *F. tularensis*.

## Materials and Methods

### Ethical statement

The protocols of all animal experiments were approved by the Institutional Animal Care and Use Committee of the National Institute of Infectious Diseases (NIID; permission no. 514005) and performed in strict accordance with the Animal Experimentation Guidelines.

### Bacterial strains

*F. tularensis* strains SCHU P9 and ∆*pdpC*, which were established in the previous report^39^, were cultured in Chamberlain’s defined medium (CDM) at 37 °C for 24 h, suspended in CDM containing 10% glycerol, and stored at −80 °C until use. Experiments with live bacterial cultures were performed in a biosafety level 3 facility following the regulations stipulated by the NIID.

### Animals

Six healthy adult male cynomolgus macaques were obtained from the animal center of the NIID. The four animals (animal no: #4548, #4549, #4550, and #4552) were imported from Vietnam and previously used in a vaccine study against simian immune-deficiency virus (SIV). These four macaques were inoculated with the recombinant BCG expressing SIV gag protein in 2001. The other two animals (#4418 and #4686), which were born at the Tsukuba Primate Research Center (National Institutes of Biomedical Innovation Health and Nutrition, Tokyo, Japan), were previously used in vaccine studies against hepatitis E virus (#4418) and influenza virus (#4686) from 2000 to 2004, respectively. These animals were housed in the animal center of the NIID after the experiments.

When used in this experiment, the average weight of the macaques was 5.6 (range, 4.5–7.6) kg. All animals were in a good physical condition and free of clinical signs of infection. During the study period, the animals were housed individually and monitored daily for signs of illness and distress. All animal experiments were performed in an animal biosafety level 3 facility.

### Immunization and challenge

The six macaques were randomly divided into three groups of two animals each. After anesthetized by intramuscular injection with ketamine hydrochloride (5 mg/kg), the two animals in Group 1 (#4550 and #4552) were subcutaneously vaccinated with 1 ml of 10^6^ CFU of strain ∆*pdpC*, whereas the four animals in Group 2 (#4418 and #4548) and Group 3 (#4549 and #4686) were subcutaneously injected with 1 ml of saline as unvaccinated controls. Three weeks later, the animals in Groups 1 and 2 were anesthetized and intratracheally challenged with 10^6^ CFU of the virulent strain SCHU P9, whereas the animals in Group 3 were anesthetized and intratracheally challenged with 10^6^ CFU of the attenuated strain ∆*pdpC* (Supplementary Table 1).

### Monitoring and blood collection

The health of the animals was monitored every day after vaccination, and appetite, water intake, behavior, appearance, and fecal production were recorded. Body temperature and body weight were measured and blood samples were collected every 2–3 dpc in accordance with the experimental scheme (Fig. 1). Animals showing severe clinical symptoms were promptly euthanized by intramuscular injection with ketamine hydrochloride (5 mg/kg) and drained of all the blood.

### Histopathological analysis

Animals were euthanized upon becoming moribund or at the end of the study and necropsies were performed in an animal biosafety level 3 facility. For histopathological and immunohistochemical analyses, the brains, spinal cords, lymph nodes, lungs, hearts, livers, spleens, kidneys, tonsils, testes, thymuses, skins, and gastrointestinal tract tissue samples were collected from each animal and immersion-fixed in 10% neutral-buffered formalin for at least 21 days to inactive all pathogens. The fixed tissues were embedded in paraffin, sectioned, deparaffinized, and stained with hematoxylin and eosin. Immunohistochemical detection of the *F. tularensis* antigen was performed on paraffin-embedded tissue sections with a polymer-based detection system, EnVision + System-HRP Labeled Polymer Anti-Mouse (Dako Denmark A/S, Glostrup, Denmark). Deparaffinized sections with no antigen retrieval reaction were treated with 0.3% H_2_O_2_ in methanol for 20 min to quench endogenous peroxidase activity. After washing with phosphate-buffered saline (PBS), sections were incubated with 5% normal rabbit serum for 5 min, followed by incubation at 37 °C for 30 min with mouse monoclonal Ab against LPS of *F*. *tularensis* (clone T14, HyTest Ltd., Turku, Finland), as the primary Ab. Sections were then reacted with a HRP-labeled polymer conjugated to goat anti-mouse immunoglobulin (Ig), then peroxidase activity was detected with 3,3′-diaminobenzidine (DAB; Sigma-Aldrich Corporation, St. Louis, MO, USA), and sections were counterstained with hematoxylin.

Double immunohistochemical staining of paraffin-embedded tissues was performed with mouse anti–*F. tularensis* LPS monoclonal Ab and a mouse anti-human CD68 monoclonal Ab (clone KP1; Dako) or a goat anti-human ACE2 polyclonal Ab (R&D Systems, Inc., Minneapolis, MN, USA). For double immunohistochemical staining of *F. tularensis* and CD68, sections were first incubated with a primary Ab against *F. tularensis* LPS. Dako EnVision^TM^ G/2 System/AP, Rabbit/Mouse (Dako) was used to detect *F. tularensis* LPS according to manufacturer’s instructions. Following heat inactivation of the Ab against *F. tularensis* LPS in 10 mM citrate buffer solution (pH 6.0) at 121 °C for 10 min, sections were incubated with Ab against human CD68 at 4 °C overnight. Dako EnVision+ System-HRP Labeled Polymer Anti-Mouse (Dako) and DAB enhanced with 0.02% cobalt chloride were used to detect the Ab against human CD68. For visualization, the sections were stained with the synthetic dye fuchsin. Nuclei were counterstained with hematoxylin.

For double immunohistochemical staining of *F. tularensis* LPS and human ACE2, the *F. tularensis* LPS antigen was first detected with the Dako EnVision+ System-HRP Labeled Polymer Anti-Mouse system. Following heat inactivation of the Ab against *F. tularensis*, LPS as described above, the sections were incubated with human ACE2 Ab at 4 °C overnight. Dako LSAB+ System–AP (Dako) was used according to manufacturer’s instructions. For visualization, the sections were stained with fuchsin, and nuclei were counterstained with hematoxylin.

### Bacterial loads in organs

Three samples of each organ (approximate 100 mg/piece) were collected from the sacrificed animals, minced, and then homogenized in RPMI 1640 medium. Serially diluted homogenates were cultured in duplicate on Eugon chocolate agar plates at 37 °C for 4 days. The number of CFU in each the minced samples was calculated as the average number of colonies in duplicate samples.

### IgG and IgM Ab responses in animals infected with *F. tularensis*

*F. tularensis*-specific Ab responses were measured using ELISA as previously reported^57^. Briefly, colonies of strain SCHU P9 were cultured on Eugon chocolate agar for 3 days, harvested, suspended in saline, adjusted to the concentration corresponding to an optical density at 600 nm (OD_600_) = 1.0, and inactivated at 100 °C for 10 min. The SCHU P9 antigen was stored at −80 °C until use. Heat-killed SCHU P9 antigen, centrifuged and diluted to 1:5 in 50 mM carbonate-bicarbonate buffer (pH 9.0) was added to the wells (100 μl/well) of a Nunc-Immuno plate (Thermo Scientific, Roskilde, Denmark), which were incubated at 37 °C overnight. After washing thrice with PBS containing 0.1% Tween 20 (PBST) to remove unbound antigen, all wells were blocked with 100 μl of PBST containing 3% skim milk at 37 °C for 1 h. After washing thrice with PBST, 50 μl of the serum samples diluted to 1:2000 in PBST containing 1% skim milk were added to antigen-coated wells in duplicate and incubated at 37 °C for 1.5 h. After washing thrice with PBST, 50 μl of anti-monkey IgM (mu chain) (goat) HRP-conjugated Ab (Rockland Immunochemicals, Pottstown, PA, USA) or anti-monkey IgG (whole molecule) HRP-conjugated anti-rabbit Ab produced (Sigma-Aldrich Corporation) diluted to 1:10,000 were added to each well, and the plates were incubated at 37 °C for 1 h. The wells were washed thrice with PBST and developed with 100 μl of 2,2′-azino-bis(3-ethylbenzothiazoline-6-sulphonic acid) ELISA HRP substrate (Roche Diagnostics Deutschland GmbH, Mannheim, Germany) at 37 °C for 30 min. The optical density at 405 nm (OD_405_) of each well was measured using a microplate reader (iMark; Bio-Rad Laboratories, Hercules, CA, USA).

### Ab responses measured by MA test

Abs in serum samples were also detected using the MA test^58^. Sera were diluted twofold, and 25 μl of each dilution was mixed with an equal volume of formalin-inactivated *F. tularensis* SCHU P9 whole-cell suspension (OD_560_ = 1.0) in the wells of a 96-well round bottom microtiter plate. The mixture was incubated at 37 °C for 18 h and checked for agglutination. *F. tularensis* immunized rabbit serum was used as a positive control, and normal rabbit serum was used as a negative control. The Ab titer in MA was expressed as the reciprocal of the highest serum dilution showing a positive response to the antigens. Agglutination at dilutions of 1:10 or greater were considered as positive for the MA Ab.

### Ab responses as determined by WB analysis

WB analysis was performed as described previously to detect *F. tularensis*-specific Ab in serum^58^. Suspensions of SCHU P9 were adjusted to OD_600_ = 1.0, mixed with the same volume of Laemmli sample buffer (Bio-Rad Laboratories), and inactivated at 100 °C for 5 min. Whole-cell lysates of *F. tularensis* SCHU P9 were subjected to sodium dodecyl sulfate-polyacrylamide gel electrophoresis (SDS-PAGE) using 5–20% precast polyacrylamide two-well gels (e-PAGEL HR, EHR-MD520L, ATTO, Tokyo, Japan). Antigens were electrophoretically transferred to an Immobilon polyvinylidene fluoride (PVDF) membrane (Millipore Corporation, Bedford, MA, USA). After incubating in PBST with 3% skim milk for 1 h at room temperature, the membrane was washed thrice with PBST for 5 min, partially dried, and cut into small strips. The sera were diluted to 1:1000 in PBST containing 1% skim milk and incubated with PVDF membrane strips at room temperature for 1 h. Then, the membrane strips were washed thrice in PBST for 5 min each, incubated with HRP-conjugated rabbit anti-monkey IgG (Sigma-Aldrich Corporation) or HRP-conjugated goat anti-monkey IgM (Rockland Immunochemicals) at a dilution of 1: 4000 at room temperature for 1 h, and then washed thrice in PBST for 5 min each. Afterward, the reactions were visualized by incubation with 0.02% DAB and 0.003% H_2_O_2_ in 0.05 M Tris-HCl buffer (pH = 7.6) for 10 min, and washed twice with distilled water. Samples were considered to contain specific Abs when the typical ladder-pattern was recognized^59^. The individual dried strips originated from the antigen transferred PVDF membrane were reacted with periodically collected serum samples of the animals and they were aligned on white papers. The grayscale images of the papers were scanned using DocuCentre-IV C2263 (Fuji Xerox, Tokyo, Japan) at superfine resolution (600 dpi) and saved as JPEG format files. The image processing was done using Photoshop CS2 (Adobe Inc, San Jose, CA, USA) to crop the images but brightness and contrast adjustment were not done. The full-length blots are shown in the figure.

### IFN-γ secreting T cells evaluated by ELISpot assay

T cells responses in peripheral blood mononuclear cells (PBMCs) were estimated using the IFN-γ, Non-Human Primate, ELISpot Kit (R&D systems, Minneapolis, MN). ELISpot assay was performed according to the manufacturer’s instruction. PBMCs collected at 0 dpc were stored at −80 °C until use. Analyses were conducted in duplicate on PBMCs from all animals. PBMCs were added to the wells of the ELISpot plate at 5 × 10^4^ cells/well in RPMI 1640 containing 10% FBS, 50 µg/ml gentamycin with and without 5 × 10^5^ of SCHU P9/well as stimulus. Cells were cultured at 37 °C, 5% CO_2_, 100% humidity for 24 h and the IFN-γ secreting T cells were evaluated. Visible spots obtained by microscope SZX10 (Olympus, Tokyo, Japan) were counted on the image files each well by ImageJ 1.51j8 software (NIH, Maryland, USA).

### Statistical analysis

All statistical analyses were performed using GraphPad Prism v5 software (GraphPad Software, Inc., La Jolla, CA, USA). Complete blood counts and concentrations of IgG and IgM were analyzed using one-way analysis of variance. A probability (*p*) value of <0.01 was considered statistically significant. The correlations at 0 dpc between Ab titers derived from ELISA data and specific IFN-γ-secreting T cells derived from ELISPOT data were plotted; then, curves were fit to a one-phase association curve using GraphPad Prism v5 software.

## Supplementary information

Supplementary Table 1
