# Supplementary material for: Protective effects of the Francisella tularensis ΔpdpC mutant against its virulent parental strain SCHU P9 in Cynomolgus macaques
Source: Sci Rep. 2019 Jun 24;9:9193. doi: 10.1038/s41598-019-45412-8 (PMC6591246; doi:10.1038/s41598-019-45412-8)
Supplement: Supplementary file 1 — Supplementary Table 1 [file 41598_2019_45412_MOESM1_ESM.docx]

**Protective effects of the *Francisella tularensis* Δ*pdpC* mutant against its virulent parental strain SCHU P9 in Cynomolgus macaques**

Deyu Tian, Akihiko Uda, Yasushi Ami, Akitoyo Hotta, Eun-sil Park, Noriyo Nagata, Naoko Iwata-Yoshikawa, AkioYamada, Kazuhiro Hirayama, Kozue Miura, Yuki Koyama, Mika Azaki, Shigeru Morikawa

| Lesions | Group 1 | |  | Group 2 | |  | Group 3 | |
| --- | --- | --- | --- | --- | --- | --- | --- | --- |
|  | #4550 | #4552 |  | #4418 | #4548 |  | #4549 | #4686 |
| Cervical lymph node | – | – |  | + ^(a,c)^ | + ^(b)^ |  | + ^(a)^ | + ^(a)^ |
| Axillary lymph node | – | – |  | + ^(a,c)^ | + ^(b)^ |  | + ^(a)^ | + ^(a)^ |
| Inguinal lymph node | – | – |  | + ^(a)^ | + ^(b)^ |  | + ^(a)^ | + ^(a)^ |
| Lung | + ^(c)^ | – |  | + ^(a,c)^ | + ^(a,c)^ |  | – | – |
| Liver | – | – |  | + ^(a)^ | – |  | – | – |
| Spleen | + ^(a)^ | ­– |  | + ^(a)^ | + ^(a)^ |  | – | – |
| Heart | – | – |  | – | – |  | – | – |
| Kidney | – | – |  | – | – |  | – | – |
| Ascites | – | – |  | + | + |  | – | – |
| Hydrothorax | – | – |  | + | + |  | – | – |

Supplementary Table 1. Macroscopic lesions observed in animals at necropsy.

+ or–indicate the presence or absence of a gross lesion.

a, enlarged; b, atrophies; c, congestion and/or hemorrhage
